# Implementing an antimicrobial stewardship medical student elective to impart good antibiotic prescribing habits early in medical training

**DOI:** 10.1017/ash.2023.522

**Published:** 2023-12-15

**Authors:** Philip W. Lam, Jennifer Lo, Jerome A. Leis, Marion Elligsen

**Affiliations:** 1 Division of Infectious Diseases, Sunnybrook Health Sciences Centre, Toronto, ON, Canada; 2 Department of Medicine, Temerty Faculty of Medicine, University of Toronto, Toronto, ON, Canada; 3 Centre for Quality Improvement and Patient Safety, University of Toronto, Toronto, ON, Canada; 4 Department of Pharmacy, Sunnybrook Health Sciences Centre, Toronto, ON, Canada

Antibiotic prescribing behavior is a complex process influenced by socio-demographic, knowledge, attitudinal, patient, and system factors.^1^ Formation of prescribing habits begins in medical school and is shaped through interactions with supervising physicians, patients, and the healthcare system.^2^ These unique experiences contribute to variability in antibiotic prescribing observed across health systems.^3,4^

Introduction of antimicrobial stewardship principles early within medical education has the potential to establish appropriate prescribing practice patterns. Although the core competencies of antimicrobial stewardship have been implemented into curricula for infectious diseases and medical microbiology trainees,^5,6^ significant opportunity exists to impart these concepts into undergraduate medical education.^7^

In 2012, a medical student clinical elective in antimicrobial stewardship was introduced at Sunnybrook Health Sciences Centre, a 627-bed Academic Health Sciences Center located in Toronto, Canada. Our antimicrobial stewardship program was established in 2009 and provides prospective audit-and-feedback to patients admitted to the intensive care, medical, and surgical wards and has resulted in sustained reductions in antimicrobial utilization.^8,9^

This two-to-four week antimicrobial stewardship elective is offered to fourth-year medical students at the University of Toronto and consists of four key components: (1) participation in prospective audit-and-feedback clinical service, (2) topic discussions with pharmacist on common infectious disease syndromes, antibiotic spectrum, pharmacokinetics, and antimicrobial stewardship concepts, (3) participation in weekly case rounds with infectious diseases and microbiology specialists, and (4) attendance of antimicrobial stewardship team meetings where system-level interventions are discussed.

On the prospective audit-and-feedback service, students work alongside stewardship pharmacists to review patients receiving broad-spectrum antimicrobials on day 3, 7, and 14. Students are taught the pertinent clinical, radiographic, and microbiologic pieces of information that form each assessment. With focus on antibiotic optimization, students present their assessments and recommendations to the infectious disease physician before communicating to the most responsible team. Their suggestions are documented in the medical chart and local antimicrobial stewardship database.

To characterize the collective experience of this medical elective, data were extracted from the antimicrobial stewardship database to describe the patients assessed by the students, types of infections assessed, and suggestions provided. Institutional research ethics review was not required because the project was deemed quality improvement and not human subject research.

Since 2012, 21 medical students have completed this rotation (0–3 per year). Descriptive data from their work on the audit-and-feedback service were available for 20 students. On average, students assessed a median of three patients per day (IQR: 2.7-–3.6). The most common infectious syndromes assessed were pneumonia (30%), urinary tract infection (16%), and intra-abdominal infection (15%). Students offered a suggestion for optimization of antibiotics in 54% of their assessments. The most common suggestions were to stop antibiotics (65%), change to an alternate antibiotic (16%), change to route of administration (16%), and change to dose (3%).

These students have gone to complete residency training in a variety of programs, including internal medicine (9), family medicine (6), general surgery (2), pediatrics (1), radiology (1), obstetrics (1), and public health and preventative medicine (1). Due to the low number of rotation evaluations received, meaningful aggregate rotation scores could not be calculated.

Providing medical students with experiential learning on an antimicrobial stewardship team is a novel method to imprint appropriate antibiotic prescribing practices. As most students will not pursue infectious diseases specialization, the goal of this rotation is to prepare them for general practice with regard to antibiotic selection, dosing, and duration for commonly encountered infections with lower case complexity.

Most undergraduate medical education antimicrobial stewardship initiatives have focused on didactic and case-based teaching.^7^ Wang and colleagues recently described a two-week antimicrobial stewardship elective consisting of didactic sessions, learning modules, case-based learning, and prospective audit-and-feedback. Through the administration of a pre-post-survey, the authors reported improvement in student confidence and knowledge in antimicrobial prescribing.^10^

Our medical elective focused on maximizing the experiential audit-and-feedback component, with each case serving as a platform to discuss approaches to antibiotic therapy, antimicrobial spectrum, and general stewardship principles. By quantifying the type of infectious syndromes assessed by medical students and recommendations provided, we hope that other antimicrobial stewardship programs can utilize this information in developing similar medical student experiences at their institution.

Immersion in the prospective audit-and-feedback role provides students with the opportunity to exercise efficient information gathering, diagnostic reasoning, and rationalization of antibiotic use. Although an infectious diseases elective rotation can help students develop a similar skill set, cases encountered tend to be more complex with more nuanced decision-making, thereby limiting the applicability to the more ordinary infections that medical trainees will encounter later in their career.

The “feedback” component of audit-and-feedback provides the student an opportunity to communicate antimicrobial recommendations to the primary services in a safe and supervised manner. This experience introduces students to the attitudinal factors (eg, fear, complacency, anxiety) which can influence antibiotic prescribing and the strategies that can be used to challenge their perspective. Developing these skills subsequently allows for self-reflection when students eventually encounter similar situations during their residency training. Student participation and observation in providing feedback on antimicrobial prescribing also fosters interprofessional collaboration, a recognized component of undergraduate medical education.

Creation of a audit-and-feedback-based antimicrobial stewardship elective for medical students represents a unique opportunity to promote judicious prescribing practices early in medical training. Further studies are needed to determine the feasibility of implementing such an elective on a larger scale and to assess the impact on long-term antimicrobial prescribing habits.

## Data Availability

Not applicable.
